# Portable Near-Infrared Technologies and Devices for Noninvasive Assessment of Tissue Hemodynamics

**DOI:** 10.1155/2019/3750495

**Published:** 2019-02-12

**Authors:** Lin Hou, Yinqiu Liu, Lixia Qian, Yucong Zheng, Jinnan Gao, Wenxing Cao, Yu Shang

**Affiliations:** ^1^North University of China, No. 3 Xueyuan Road, Taiyuan 030051, China; ^2^Shanxi Dayi Hospital, No. 99 Longcheng Street, Taiyuan 030032, China; ^3^University of Electronic Science and Technology of China, No. 2006 Xiyuan Street, Chengdu 611731, China

## Abstract

Tissue hemodynamics, including the blood flow, oxygenation, and oxygen metabolism, are closely associated with many diseases. As one of the portable optical technologies to explore human physiology and assist in healthcare, near-infrared diffuse optical spectroscopy (NIRS) for tissue oxygenation measurement has been developed for four decades. In recent years, a dynamic NIRS technology, namely, diffuse correlation spectroscopy (DCS), has been emerging as a portable tool for tissue blood flow measurement. In this article, we briefly describe the basic principle and algorithms for static NIRS and dynamic NIRS (i.e., DCS). Then, we elaborate on the NIRS instrumentation, either commercially available or custom-made, as well as their applications to physiological studies and clinic. The extension of NIRS/DCS from spectroscopy to imaging was depicted, followed by introductions of advanced algorithms that were recently proposed. The future prospective of the NIRS/DCS and their feasibilities for routine utilization in hospital is finally discussed.

## 1. Introduction

Many diseases would lead to abnormal status in microvasculature blood flow, oxygenation, and oxygen metabolism [1, 2]. For example, ischemic stroke causes reduction in both blood flow and oxygenation [3]. Malignant tumor leads to higher blood flow, higher oxygen consumption rate, and lower oxygen status [4, 5]. For the people with obstructive sleep apnea, large fluctuations in microvasculature blood flow and total hemoglobin concentration were discovered [6].

There are few technologies currently available to noninvasively monitor the oxygen status in tissue. PO_2_ is a technology capable of quantifying the oxygen pressure via a tiny needle [7]. This invasive pattern of measurement, however, is unfavorable to healthy population. As a conventional technology for tissue blood oxygenation measurement including the oxy-, deoxy-, total hemoglobin concentration ([HbO_2_], [Hb], and THC), and oxygen saturation (StO_2_), near-infrared diffuse optical spectroscopy (NIRS) has been developed for more than four decades, since 1970s [8]. NIRS oximeter was often utilized in clinical and physiological studies [8–10] for detecting the relative deep tissue (up to several centimeters), owing to the low tissue absorption in near-infrared (NIR) range (650 to 900 nm). However, the oxygenation alone only reflects the static balance between the oxygen supply and consumption [11]. Blood flow is such a parameter that elucidates how fast and efficient the oxygen is carried in the blood and supplied to the tissue. Studies show that blood flow is highly sensitive to pathophysiological alteration [12], thus could be a potential indicator for early detection of many diseases. Additionally, the blood flow reflects the reactivity patterns of vascular vasodilatation [3], thus assisting in evaluation of vessel functions. Therefore, investigation of blood flow, as well as its correlations with pathophysiological consequences, has significant implications for diagnosis and medications. Furthermore, the combination of tissue blood flow and oxygenation allows for estimate of the oxygen metabolic rate [11, 13], a valuable parameter closely associated with tissue pathology.

At present, the low-cost modalities to noninvasively and fast measure the blood flow in deep tissue are not established. Methods currently available for assessment of blood flow include Doppler ultrasound [14], laser Doppler [15], and perfusion-weighted magnetic resonance imaging (perfusion MRI) [16]. Doppler ultrasound can only assess the blood velocity in single and large vessel [14, 17]. Many diseases, however, are relevant to the abnormal blood flow in microvasculature level rather than macrovasculature level [1, 2]. While perfusion MRI can provide the microvasculature blood flow at high spatial resolution, the high cost and low mobility of MRI instrument preclude its wide use for routine disease screening. Laser Doppler can only detect blood flow at superficial tissue (∼1 mm depth) [18], thus unable to probe the diseases mostly originated from deep tissues. In summary, the routine use of the above techniques is limited due to availability, expense, and the difficulty of making continuous measurements.

In recent years, a dynamic NIRS technology, namely, diffuse correlation spectroscopy (DCS), has gained rapid development [19–23]. DCS quantifies the speckle fluctuations of diffuse light, which are sensitive to the motion of red blood cells in the microvasculature, thus permitting fast assessment of blood flow in deep tissues (up to several centimeters).

The article will briefly describe the technology and devices for NIRS and DCS, as well as their applications to physiological studies and clinic. The extension of NIRS from spectroscopy to imaging was depicted, followed by introductions of advanced algorithms that were recently proposed. The future prospective of the NIRS and DCS and their feasibilities for routine use in hospital was finally discussed.

## 2. NIRS Principle and Devices

When being injected into the biological tissues that are considered as turbid medium, the near-infrared light transports through the medium in “diffusive” manner [2], due to tissue absorption (mainly red blood cells) and scattering (mainly organelles and mitochondria) [1, 24]. The aim of NIRS technology is to separate the tissue absorption from scattering, through which the hemoglobin concentration is derived. Over the years, two approaches, namely, the time-resolved spectroscopy [25, 26] and frequency-modulated spectroscopy [27, 28], have been developed to account for this separation. For the time-resolved approach, a pulsed laser (a few of picoseconds) is adopted and the time-of-flight of photons is recorded by a digital time-correlated single photon counting (TCSPC) board. For frequency-modulated approach, the light is modulated at high frequency (around 100 MHz) [29], so that the phase of modulated light escaping out from the tissue could be traced.

Both these two approaches were commercialized with numerous applications [30], particularly for neurological studies. Either the time-resolved or frequency-modulated approach, however, requires complicated hardware to manipulate the light, substantially increasing the cost of instrument.

In practice, the NIRS was often simplified by using continuous-wave (CW) light instead of pulsed or frequency-modulated light. In CW oximeter, the tissue scattering (*μ*s) was assumed to be a constant number and only the relative change of tissue absorption coefficient needs to be determined by the modified Beer–Lambert law.

(1) $$\begin{array}{c} \ln\left(\frac{I\left(t_{0}\right)}{I\left(t\right)}\right)=\Delta \mu_{\text{a}}\left(\lambda \right)\cdot L=\left(\varepsilon_{\text{HbO}}\left(\lambda \right)\Delta \left[{\text{HbO}}_{2}\right]+\varepsilon_{\text{Hb}}\left(\lambda \right)\Delta \left[\text{Hb}\right]\right)\cdot L, \end{array}$$

where *I*(*t*_0_) and *I*(*t*) are the light intensity detected at time *t*_0_ and *t*, respectively; Δ*μ*_a_(*λ*) is the relative change of the absorption coefficient at wavelength *λ*; and *L* is the mean photon length between a specific source-detector (S-D) pair. By collecting the light at two wavelengths, the oxygenation changes can be calculated as [31–33]

(2) $$\begin{array}{c} \Delta \left[{\text{HbO}}_{2}\right]=\frac{\varepsilon_{\text{Hb}}\left(\lambda_{1}\right)\Delta \mu_{a}\left(\lambda_{2}\right)-\varepsilon_{\text{Hb}}\left(\lambda_{2}\right)\Delta \mu_{a}\left(\lambda_{1}\right)}{\varepsilon_{\text{Hb}}\left(\lambda_{1}\right)\varepsilon_{\text{HbO}}\left(\lambda_{2}\right)-\varepsilon_{\text{HbO}}\left(\lambda_{1}\right)\varepsilon_{\text{Hb}}\left(\lambda_{2}\right)},\\ \Delta \left[\text{Hb}\right]=\frac{\varepsilon_{\text{HbO}}\left(\lambda_{2}\right)\Delta \mu_{a}\left(\lambda_{1}\right)-\varepsilon_{\text{HbO}}\left(\lambda_{1}\right)\Delta \mu_{a}\left(\lambda_{2}\right)}{\varepsilon_{\text{Hb}}\left(\lambda_{1}\right)\varepsilon_{\text{HbO}}\left(\lambda_{2}\right)-\varepsilon_{\text{HbO}}\left(\lambda_{1}\right)\varepsilon_{\text{Hb}}\left(\lambda_{2}\right)}, \end{array}$$

where Δ[HbO_2_] and Δ[Hb] are the relative change of oxy- and deoxy-hemoglobin concentration and *ε*_HbO_(*λ*) and *ε*_Hb_(*λ*) are their extinction coefficients, respectively.

**Figure 1:**
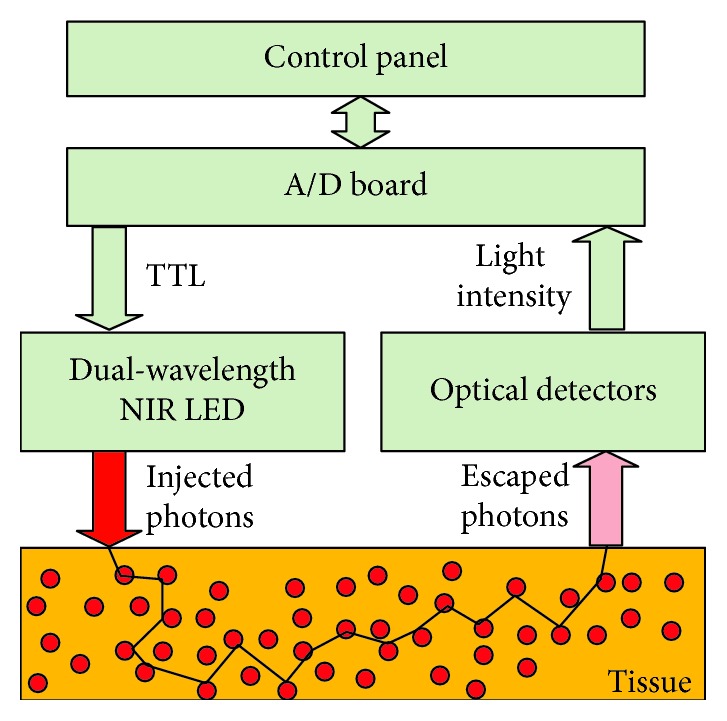
Graphic illustration of a NIRS tissue oximeter.

Figure 1 illustrates a typical scheme of CW oximeter. Briefly, a dual-wavelength LED at NIR range was controlled by transistor-transistor logic (TTL) signals, injecting the photons at each wavelength into the tissue alternately. The escaped photons were collected in parallel by all optical detectors and transferred to the computer, wherein the modified Beer–Lambert law was applied, yielding time-course oxygenation data.

The reason for using CW light as the source light of NIRS technology is that the tissue scattering (majorly contributed from organelles and mitochondria) changes much less than the tissue absorption in most of physiological status. Thus, the tissue blood oxygenation would still been properly extracted by the CW tissue oximeter. Moreover, this low-cost device could minimize the cross-talk between the absorption and scattering.

The CW tissue oximeter was also commercialized by different manufacturers (e.g., NIRO, Hamamatsu Inc.; INVOS, Somanetics Inc.) and has various clinical applications. At CW working mode, the absolute value of tissue absorption coefficient (*μ*_a_) could be extracted by the near-infrared light collected from multiple source-detector (S-D) separations, according to the theory of spatial-resolved spectroscopy [34]. This theory permits generation of a slope through multiple S-D separations of optical data, from which the tissue absorption will be determined. In general, more S-D separations perform better in calculating absorption coefficient (*μ*_a_) with stable and accurate values. For example, four S-D separations (2.0 to 3.5 cm) were used in a commercial tissue oximeter (OxiplexTS, ISS Inc). On the other hand, however, more S-D separation leads to higher cost of instrumentation and increases the size of optical probe. Hence, two S-D separations, i.e., the minimal number to determine a slope, were used more frequently in commercial instruments (e.g., NIRO, Hamamatsu Inc.; INVOS, Somanetics Inc.).

Despite the wide use of CW light, it is worthy to note that the tissue absorption coefficient (*μ*_a_) and reduced scattering coefficient (*μ*s′) cannot be uniquely determined from the CW tissue oximeter alone, regardless of the number of S-D separations being used [35]. To separate these two coefficients, time-resolved spectroscopy or frequency-modulated spectroscopy must be adopted.

## 3. DCS Principle and Devices

**Figure 2:**
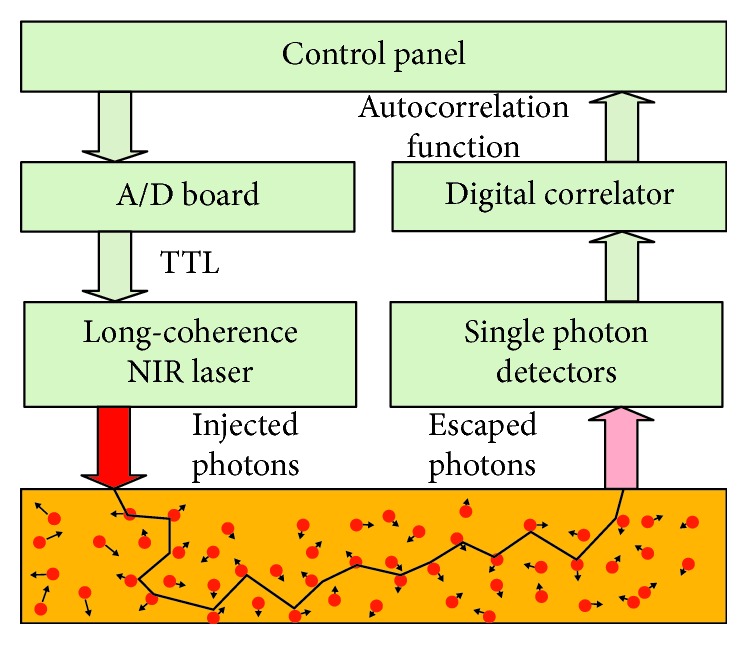
Graphic illustration of a tissue flowmeter.

DCS is originated from the technology of dynamic light scattering [36, 37], in which single scattering event is allowed within thin biological sample. To account for *in vivo* measurement in bulk tissues or organs, advanced mathematical procedures were proposed to model the multiple scattering event, namely, diffuse correlation spectroscopy (DCS) [20, 38], or diffusing wave spectroscopy (DWS) [39, 40]. As depicted in Figure 2, a long coherence length (>5 m) laser in near-infrared range injects photons into the tissue via optical fiber. The photons travel through the tissue and are scattered back to the detector via a single mode fiber. There are fluctuations in the intensity of escaped photons, which is due to the moving of scatters (mainly red blood cells) in the microvasculature system. A digital correlator takes the detector output and uses photon arrival times to compute the light intensity autocorrelation function. From the normalized intensity autocorrelation function, the electric field temporal autocorrelation function *G*_1_(*τ*), which satisfies the correlation diffusion equation in turbid medium [20, 38], is calculated. Approximately, the normalized electric field temporal function *g*_1_(*τ*) at early delay time decays exponentially with *τ* (delay time). The blood flow index (BFI) information is extracted by fitting the curve of temporal function *g*_1_(*τ*) whose decay rate depends mainly on the motion of red blood cells [20, 21, 41].

The current optical probes for DCS/DCT are designed with source and detector fibers confined by a flat foam pad, restricting the use of this technology for tissues with different geometries. Moreover, the requirement in temporal/spatial resolution varies across different tissues and organs.

As a relative new technology, there are commercial devices for DCS (e.g., Neuro-Monitor, HemoPhotonics Inc., Spain). Similar to tissue oximeter, this commercial DCS/DCT device is primarily utilized for neurological function evaluation, limiting its application for other diseases.

## 4. Integrated System and Hybrid Optical Probe

Either the NIRS or DCS, when being used alone, could provide only single hemodynamic parameter (oxygenation or blood flow). At early time, the NIRS and DCS devices were applied, respectively, to the biological tissues, in order to obtain comprehensive hemodynamic information. Under this framework, however, it is difficult to measure the oxygenation and blood flow from the same tissue because the NIRS and DCS probes cannot be physically overlapped. More importantly, since the two devices are controlled independently, the source light from one device interferences with the signal collection by another device, if the two probe are placed closely on the tissue. In recent years, attempts have been made to combine the DCS with NIRS devices to form an integrated system, for simultaneous measurement of blood flow and oxygenation at microvasculature level, also permitting estimate of oxygen metabolic parameters (e.g., oxygen consumption rate) [11, 42]. Such integrated NIRS/DCS system for tissue oxygen consumption monitoring is already commercially available (MetaOx, ISS Inc., USA). Prior to the commercial products, the integrated system has been constructed by various research groups.

**Figure 3:**
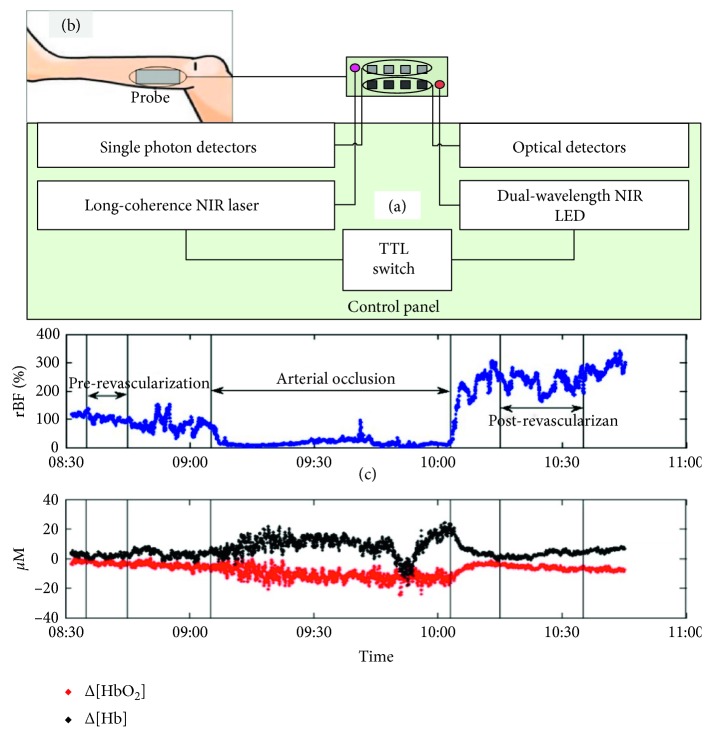
Graphic illustration of the integrated optical system (a) and the hybrid optical probe on human lower limb (b). The application of this system to simultaneously monitor the blood flow and oxygenation changes during arterial revascularization is exhibited (c).

Figure 3 shows a typical integrated optical system combing the tissue oximeter and flowmeter. Briefly, the dual-wavelength NIR LED (oximeter) and the long-coherence NIR laser are turned on/off by the TTL switch (Figure 3(a)), launching the photon into biological tissues alternately. The photons scattered back from the tissue are collected by either optical detectors (oximeter) or single photon detectors (flowmeter). In this pattern, the two devices work on/off in sequential pattern.

To comply with the integrated system, one needs a hybrid optical probe, containing all source and detector fibers in place. This configuration of fiber setup ensures the blood flow and oxygenation information measured by DCS and NIRS to cover mostly the same volume in the tissue. As mentioned earlier, the sources of DCS and NIRS are turned on/off sequentially, in order to avoid the light interference between the two technologies.

For construction of hybrid optical probe, a foam pad is used to house both NIRS and DCS fibers (Figure 3(b)) that are originally distributed in two separated pads. Within the pad, the fibers are arranged to form multiple-distance source-detector pairs for both NIRS and DCS measurements. This all-in-one probe has many advantages, such as compact size, easy to tape, and enable detection of blood flow and oxygenation in an overlapped tissue volume, as stated earlier, making it possible to estimate the metabolic parameters (e.g., the oxygen consumption rate). Moreover, the multiple separations provide hemodynamic information from different layers of tissues (e.g., scalp, skull, and brain cortex), which can be separated by using the advanced algorithms.

The design of optical probe would be adaptable to different tissues or organs. For the brain studies, there is little deformation on the head when subject to contact pressure. Hence, the materials with good elasticity and stiffness (e.g., silicon glue and polymer foam) are often used to house the S-D fibers, which would adapt the small curvature of the human head in contact pattern. For measurement of the breast tumor in which large tissue deformation occurs, noncontact pattern of measurement is preferred in order to adapt to the large curvature of breast. For such clinical applications, a lens system is designed to focus the source and detector on the tissue surface [4], permitting noncontact collection of optical signals. To minimize the instrumentation cost, the lens system will be controlled to scan over the tissue surface, greatly reducing the number of optical components (laser and detectors) [4].

The integrated NIRS system and the hybrid optical probe, depicted above, have advantages of high-throughput, portability, and easy-to-use, which have high flexibility to be used in different tissue type, disease category, and clinical setting. The successful carrying out of the hemodynamic measurements promote the translation of DCS/DCT into clinic, making it possible to routinely use this technology for early detection and therapeutic evaluation of a variety of diseases.

Table 1 lists the physiological or clinical studies wherein the integrated NIRS/DCS instrument and hybrid probe were used. Among these, a small portion of studies reports the improvements on the optical instrumentation or probe design. For example, Shang et al. [32] combined a commercial frequency-domain NIRS oximeter and a dual-length DCS flowmeter to form an integrated system. Accordingly, a hybrid probe that houses the NIRS fibers and DCS fibers in the same foam was designed. In another study, Li et al. [62] designed a noncontact probe which focuses the source and detector light on the surface of the target tissue via lens system. This probe permits simultaneous monitoring of the blood flow and oxygenation without contact with the tissues. This noncontact pattern of measurement is suitable to be used in deformable tissues such as human breast. Both contact and noncontact systems were generally validated through the manipulation protocol of temporary cuff occlusion on the limb, which reduces the blood supply to the skeletal muscle where the blood flow and oxygenation are measured.

In addition to the instrumentation improvements as mentioned above, the majority of studies using the integrated system focused on the physiological or clinical applications. For the physiological studies, a variety of experimental manipulation were designed to challenge the target tissues, such as the hypercapnia to rat brain [20], cortex activation to human brain [43], plantar flexion or handgrip exercise to human skeletal muscles [58, 66], posture change to human brain [53, 56, 65], and the hypothermic to swine brain [68].

These physiological manipulations alter the amount of tissue hemodynamics (blood flow oxygenation and oxygen consumption rate), making it convenient to detect the potential defects or diseases (e.g., aging, autoregulation impairment, and vasovagal syncope).

For the clinical or preclinical studies, the integrated system was primarily used to probe ischemic diseases or tumor. For these purposes, animal models were often used to create the relevant diseases, including the cerebral ischemia on rat and mouse [3, 42], hindlimb ischemia on the mouse [52], and melanoma tumor on the mouse [47]. The outcomes derived from the animal studies would assist the understanding of pathophysiological mechanism and improvements of therapeutic approaches. In addition to the animal models, the integrated system was also directly applied to clinic, due to the advantages of noninvasiveness and easy-to-use. For example, Sunar et al. [47] and Dong et al. [12, 57] longitudinally monitored the blood flow and oxygenation changes in the head/neck tumor over weeks of chemoradiation therapy and found these hemodynamic changes were associated with pathophysiological outcomes. Yu et al. [46] and Zhou et al. [50] assessed the hemodynamic changes in prostate and breast tumors during photodynamic therapy and chemotherapy, respectively. Durduran et al. [49] investigated the hemodynamic responses to posture change in the stroke patients and found that the response pattern was significantly affected by the diseases. Shang et al. [33] and Yu et al. [55], on the other hand, evaluated the integrated system for intraoperative monitoring of the carotid and femoral arterial surgeries, respectively. The experiment results verified the high sensitivity of the integrated NIRS/DCS technology for ischemic detection during the vascular surgeries.

As a graphic example, Figure 3(c) shows the muscle hemodynamics (blood flow and oxygenation) during arterial vascularization [55]. It is clearly seen that the arterial clamping causes acute ischemia and hypoxia on the muscle tissue, i.e., decrease in blood flow/Δ[HbO_2_] and increase in Δ[Hb]. Following the release of cuff occlusion, there is hyperemic response, elevating the blood flow to a peak value. Thereafter, both the blood flow and oxygenation recover gradually towards their baseline. Additionally, the postrevascularization improvements in tissue hemodynamics were detected. This study demonstrates the high sensitivity of NIRS/DCS in monitoring the intraoperative interventions as well as the acute therapeutic assessments.

In addition, there are reports of hemodynamic assessment on other diseases, including the congenital heart defects [51, 64], premature [13], critically brain-injured [54], fibromyalgia [11], and peripheral artery disease [60, 67]. Those clinical studies validated the wide usage of integrated NIRS/DCS system for various diseases.

## 5. Imaging Modalities for NIRS and DCS

The NIRS and DCS provide time variation of oxygenation and blood flow, respectively. In order to obtain the spatial contrast of the tissue hemodynamics, there is a need to extend the NIRS and DCS from spectroscopy to tomography, termed as diffuse optical tomography (DOT) and diffuse correlation tomography (DCT), respectively. The DOT/DCT share the same physical principle of NIRS/DCS, except that far more S-D pairs are needed to perform image reconstruction. As reported in literature, a high-density DOT system consists of 96 sources and 92 detectors, providing the optical measurements from more 1200 S-D pairs [69]. Similarly, a representative DCT device consists of 2 laser diodes and 15 APD and with 15 steps of line scanning, generating a total of 450 autocorrelation *g*_1_(*τ*) curves [70]. In addition to the hardware configuration, the algorithms adopted for image reconstruction is critical in determining imaging quality of blood flow or oxygenation, which will be discussed in Section 6. Apparently, a large number of optical components (laser, detector, etc.) are required for DOT or DCT, substantially elevating the cost of instrumentation. A few efforts have been made to lower down the physical S-D measurements. For example, the line or rotated scanning could efficiently reduce the number of optical components but needs the optical probe to operate in noncontact pattern. The noncontact measurement, however, is susceptible to the motion artifacts and ambient light. The optical switch, which enables sharing the laser or detector among multilocation measurements, could be a good alternative option to save the instrument cost. Nevertheless, a number of hardware source and detector pairs are still needed for the imaging of tissue hemodynamics, making the instrument no longer portable. Thus, the hemodynamic imaging is not the focus of this article.

## 6. Advanced Algorithms for NIRS and DCS

Apart from instrumentation, the reconstruction algorithm of DCS/NIRS also contributes greatly to the accuracy and robustness of oxygenation and blood flow values, which is highly crucial to the detective sensitivity of diagnostic outcomes [31, 71]. For DCS, as stated earlier, the conventional algorithm is based on the analytical solution of a partial differential equation (PDE) [20, 21, 41]. However, the analytical solution of PDE is dependent on tissue geometry and usually has complicated mathematical forms. For the purpose of easy implementation, the tissue geometry is often assumed as semi-infinite. There are also analytical solutions to PDE for other regular tissue geometries, such as cylinder and sphere [41, 72]. However, those solutions are rarely adopted for practical applications, due to the complicated and tedious computations. In recent years, a finite element method (FEM) for DCT was developed based on similar approach for NIRS [70]. Although the FEM was proved to be efficient in reconstruction of flow value in tissue with complicated geometry, it is difficult to fully utilize autocorrelation data, thus susceptible to data noise.

For sufficient use of the DCS/DCT data, a mathematical approach, namely, the Nth-order linear algorithm (i.e., NL algorithm), was developed for extracting BFI value. Unlike the analytical solution or FE method, the NL algorithm does not seek for PDE solution. Instead, it was originated from a combination of a Nth-order Taylor polynomial and the integral form of g_1_(*τ*). After a series of mathematical procedures, the BFI is extracted by iteration of linear regressions, ultimately reaching the following expressions [73]:

(3) $$\begin{array}{c} g_{1}\left(\tau ,j\right)-1=\tau \overset{n}{\underset{i=1}{\sum }}A\left(i,j\right)\alpha D_{B}\left(i\right), \end{array}$$

(4) $$\begin{array}{c} g_{1}\left(\tau ,j\right)-1-\overset{N}{\underset{k=2}{\sum }}\frac{{\sum_{p=1}^{Q}w\left(p,j\right)\left(-2\sum_{i=1}^{n}k_{0}^{2}\left(i\right)\alpha D_{B}\left(i\right)s\left(i,p,j\right)\mu_{s}^{\prime }\left(i\right)\right)}^{k}}{k!}\tau^{k}=\tau \overset{n}{\underset{i=1}{\sum }}A\left(i,j\right)\alpha D_{B}\left(i\right). \end{array}$$

Equations (3) and (4) are called first-order and Nth-order linear (NL) algorithm, respectively. Here, *α*  *DB*(*i*) is the BFI of the *i*th tissue component; *w*(*p*, *j*) is the normalized weight of photon package collected by *j*th detector; and *s*(*i*, *p*, *j*) is the path length of *p*th photon package traveled within *i*th tissue component and collected by *j*th detector. The coefficient of matrix *A*(*i*, *j*) has the following expression:

(5) $$\begin{array}{c} A\left(i,\;\;j\right)\;=\;\overset{Q}{\underset{p=1}{\sum }}-2w\left(p,\;\;j\right)k_{0}^{2}\left(i\right)s\left(i,\;\;p,\;\;j\right)\mu_{s}^{\prime }\left(i\right). \end{array}$$

The key parameter contained in equations (3)–(5) is *s*(*i*, *p*, *j*), which is determined by the tissue geometry and heterogeneity, indicating that both of these two features are fully taken into consideration in the NL algorithm. The accuracy of the NL algorithm on various geometries and tissue heterogeneity was validated in DCS, through computer simulations as well as animal experiment [73, 74].

The similar procedure for heterogeneous tissue is also applicable to NIRS, leading to the following equations to calculate the changes of chromophore concentration [31, 75]:

(6) $$\begin{array}{c} \left(\begin{array}{c} {\text{OD}}_{1}\\ \ldots \\ {\text{OD}}_{N} \end{array}\right)=\left(\begin{array}{ccc} s_{11} & \ldots & s_{1M}\\ \ldots & \ldots & \ldots \\ s_{N1} & & s_{NM} \end{array}\right)\left(\begin{array}{c} \Delta \mu_{a,1}\\ \ldots \\ \Delta \mu_{a,M} \end{array}\right),\;\left(M\leq N\right), \end{array}$$

where OD_*i*_ is the optical density from *i*th S-D measurement; *s*_*ij*_ is the photon path length within *j*th tissue measured from *i*th S-D pair; *M* and *N* are the numbers of tissue and S-D pair, respectively; and Δ*μ*_a,*j*_ is the relative change of the absorption coefficient at *j*th tissue. For convenience in matrix operation, equation (6) can be abbreviated as

(7) $$\begin{array}{c} \text{OD}=s\Delta \mu_{\text{a}}. \end{array}$$

Here, *s* is the matrix of photon path length. When *M* = *N*, the changes of absorption coefficient can be calculated according to the following equation:

(8) $$\begin{array}{c} \Delta \mu_{\text{a}}=s^{-1}\text{OD.} \end{array}$$

It is possible to extract the hemodynamic information at higher accuracy by using the optical signals from more measurements (i.e., *N* > *M*). In this situation, the least-square fitting method can be used to calculate the changes of absorption coefficient at different tissues:

(9) $$\begin{array}{c} \Delta \mu_{\text{a}}={\left(s^{T}s\right)}^{-1}s^{T}\text{OD}. \end{array}$$

Due to the correlation of optical signals among different S-D pairs, the matrix *s* is often ill-posed, i.e., susceptible to the data noise. The noise influence on oxygenation measurement could be partially alleviated by introducing regularization approaches. One of the widely-used approaches is Tikhonov regularization, with which the final form of hemoglobin calculation is the following:

(10) $$\begin{array}{c} \Delta \mu_{\text{a}}={\left(s^{T}s+\alpha^{2}I\right)}^{-1}s^{T}\text{OD}. \end{array}$$

Here, *α* is the constant number controlling the balance between the equation fidelity and solution robustness.

## 7. Clinical Applications

Over the years, the NIRS and DCS for tissue oxygenation and blood flow measurements have been extensively validated to other modalities and been translational to various physiological and clinical studies, such as assessment of autoregulation capability [56], therapeutic monitoring of ischemic stroke [33], and early detection of tumors [4]. These applications are summarized in Table 1 and briefly reviewed in Section 4. Some representative applications are introduced with details in the following subsections.

### 7.1. Assessment of Autoregulation Capability

Studies have shown that many diseases, such as ischemic stroke, obstructive sleep apnea, vasovagal syncope, or Alzheimer's disease, may cause deficit in autoregulation capability, which could be assessed by alteration of both blood pressure and cerebral hemodynamics. For example, change of posture could alter both blood pressure and cerebral hemodynamics. However, the changing pattern of cerebral hemodynamics in patients, dependent on the autoregulation capability, may differ significantly from those in healthy subjects, from which we can detect the neurological deficit. The technologies of NIRS and DCS were well established to measure the cerebral hemodynamics, and the blood pressure could be longitudinally assessed by a finger plethysmograph, at the pulse frequency (∼1 Hz). The physiological protocols to induce the cerebral hemodynamics include enforced breathing at 0.1 Hz, cuff occlusion on both side of lower limbs, posture transition from squatting to standing, and the head-up-tilting (HUT). Among these, the 70-degree HUT was adopted widely in physiological studies. For most of the people, it was found that there are spontaneous low frequency oscillations (LFOs) around 0.1 Hz in some physiological parameters including the blood pressure and hemodynamics, and the LFOs could be affected by the impairment in autoregulation capability. For rapid and noninvasive assessment of the autoregulation capability, a hybrid near-infrared diffuse optical instrument was constructed to simultaneously detect LFOs of cerebral blood flow (CBF) and oxygenation ([HbO_2_], [Hb], and THC) [56], and a finger plethysmograph was used to detect mean arterial pressure (MAP). The coherence outcomes of LFOs between MAP and each of the hemodynamic variables show that the CBF, [HbO_2_], and THC were reliable hemodynamic parameters in detecting LFOs [56], which has clinical implication in early diagnosis of cerebral impairment caused by ischemic stroke, obstructive sleep apnea, vasovagal syncope, or Alzheimer's disease.

### 7.2. Therapeutic Monitoring of Ischemic Stroke

During many head and neck surgeries, such as carotid endarterectomy, the major arteries, such as common carotid artery, internal carotid artery, and middle cerebral artery, are temporarily occluded for blooding prevention and operating convenience. On the other hand, this procedure increases the risk of ischemic stroke. Therefore, real-time monitoring of cerebral hemodynamics is critical for minimizing the intraoperative stroke and acute assessment of surgical outcomes. For this purpose, the DCS and NIRS were combined in a portable device, namely, DCS flow-oximeter [32]. As a pilot study of intraoperative assessment, two fiber-optic probes were taped on both sides of forehead for cerebral hemodynamic measurements [33]. Meanwhile, the brain wave was monitored by electroencephalogram (EEG). The cerebral blood flow and oxygenation were simultaneously monitored on twelve patients throughout entire period of carotid endarterectomy. The results verified the feasibility and sensitivity of NIRS and DCS in probing transient cerebral ischemia due to arterial clamping and postsurgical hyper perfusion [33]. More importantly, the comparison outcomes show that the CBF responses to arterial clamping, measured by DCS, were faster, larger, and more sensitive than EEG responses. This study suggests the potential of NIRS/DCS technologies for routine use in surgical rooms.

### 7.3. Early Detection of Breast Tumors

The blood flow, which reflects the dynamic demands of oxygen in tissue, is highly sensitive to the growth of the tumor with extraordinary high metabolic rate. Hence, monitoring of microvasculature blood flow, along with oxygenation measurement, will probe the malignant tumor earlier than the morphological imaging modality such as ultrasound, CT, or MRI. The portable NIRS technology has been used to early diagnosis a few cancers, majorly, the breast tumors.

In an earlier study, a hand-held optical probe, containing four S-D pair fibers, was used to scan over the breast horizontally and vertically [5]. The optical data were transferred into a DCS device for extracting the blood flow index (BFI) at different locations. In the women with breast cancer, a high contrast in BFI was found between malignant tumor and the normal tissue, demonstrating the high sensitivity of DCS in detecting the abnormal blood flow caused by the cancer. However, the breast tissue is soft and susceptible to contact pressure. Hence, it is difficult to obtain the true BFI values in natural posturer. Recently, a noncontact probe is constructed, through which the light emitted into (or escaped out from) the tissue is delivered by the lens system [76], rather than directly contacting with the skin. The noncontact pattern of measurement efficiently avoids the breast deformation caused by the contact pressure. Moreover, the motor-controlled line scanning system permits data acquisition from a large number of S-D pairs, making it possible to reconstruct the breast tumor imaging [70]. Similar to the contact-measurement study mentioned above, the imaging from the women with malignant tumor exhibits high spatial contrast (5.9- and 10.0-fold) between the malignant tumor and normal tissue [4].

These studies indicate the potential of NIRS/DCS systems for noninvasive and early diagnosis of the breast tumors, which is greatly beneficial to the healthcare of middle and aging women.

In addition to the clinical applications mentioned above, the NIRS/DCS has been widely translated to assess the tissue hemodynamic status for other diseases or medical interferences, including the peripheral artery disease (PAD) [60], fibromyalgia [11], radiation therapy on head/neck tumor [12], and tissue transfer flaps [77]. Due to the length limit, the readers are encouraged to see references for more details.

## 8. Challenges and Future Prospective

Although some NIRS commercial products have been utilized for physiological and disease studies, there are some challenges for its wide translation to clinic. For example, the absolute value of oxygenation parameters ([HbO_2_], [Hb], THC, and StO_2_) varies over a large range in healthy population, making it difficult to set a threshold to separate the diseased tissues and normal tissues. In practice, therefore, the oxygenation contrast, either temporally or spatially, was often used for abnormal detection. Likewise, the standard procedure to detect abnormal tissue with DCS flow measurement has not been established. Besides, a few factors, such as the ambient noise, the curvature of tissue geometry, and the contact pressure between optical probe and tissue surface, will affect the NIRS/DCS technology for hemodynamic measurement.

In summary, this article provides a review of the static and dynamic NIRS technologies in comprehensive aspects, including the physical principle, the instrumentation, the algorithms for hemodynamic calculation, and the applications in physiological and clinical measurement. Over four decade development, the NIRS technology has demonstrated excellent feasibility in exploring the microvasculature oxygen kinetics and pathophysiological mechanism. The success of hemodynamic measurement greatly promoted the translation of NIRS technology to clinic. However, the accuracy and efficiency of this technology for early disease detection and therapeutic evaluation is heavily dependent on the tissue type, disease category, and clinical environment. Thus, there are still urgent needs to develop utility-driven strategy for routine application of NIRS/DCS in clinic. Ultimately, we anticipate that the portable, inexpensive NIRS/DCS systems will be widely commercialized for routine utilization in clinical rooms and patient's bedside.

## Figures and Tables

**Table 1:** Studies wherein the integrated NIRS/DCS instrument and hybrid probe were utilized.

Year	Ref. no.	Authors	Target tissue	Measured variables	Physiological manipulation/diseases
2001	[20]	Cheung et al.	Rat brain	BFI, hemoglobin, StO_2_	Hypercapnia
2003	[42]	Culver et al.	Rat brain	BFI, hemoglobin, StO_2_ oxygen consumption rate	Ischemia
2004	[43]	Durduran et al.	Human brain	BFI, hemoglobin change, oxygen consumption rate	Cortex activation
2005	[44]	Yu et al.	Human skeletal muscle	BFI, hemoglobin, StO_2_ oxygen consumption rate	Plantar flexion exercise
2006	[45]	Sunar et al.	Human tumor	BFI, hemoglobin, StO_2_	Head/neck tumor
2006	[46]	Yu et al.	Human tumor	BFI, StO_2_	Prostrate tumor
2007	[47]	Sunar et al.	Mouse tumor	BFI, StO_2_	Melanoma tumor
2007	[48]	Zhou et al.	Human tumor	BFI, hemoglobin	Breast tumor
2009	[32]	Shang et al.	Human skeletal muscle	BFI, hemoglobin change	Cuff occlusion on upper limb
2009	[49]	Durduran et al.	Human brain	BFI, hemoglobin, StO_2_	Stroke
2009	[50]	Zhou et al.	Piglet brain	BFI, hemoglobin, StO_2_	Closed head injury
2010	[51]	Durduran et al.	Human infant brain	BFI, hemoglobin oxygen consumption rate	Congenital heart defects
2010	[52]	Mesquita et al.	Mouse skeletal muscle	BFI, StO_2_ oxygen consumption rate	Hindlimb ischemia
2010	[13]	Roche-Labarbe et al.	Human infant brain	BFI, hemoglobin, StO_2_ oxygen consumption rate	Premature
2010	[53]	Edlow et al.	Human infant brain	BFI, hemoglobin	Posture change
2010	[54]	Kim et al.	Human brain	BFI, hemoglobin change	Critically brain-injured
2011	[3]	Shang et al	Mouse brain	BFI, hemoglobin change	Ischemia
2011	[55]	Yu et al.	Human skeletal muscle	BFI, hemoglobin change	Ischemia
2011	[33]	Shang et al.	Human brain	BFI, hemoglobin change	Ischemia
2012	[56]	Cheng et al.	Human brain	BFI, hemoglobin change	Posture change
2012	[57]	Dong et al.	Human tumor	BFI, hemoglobin change	Head/neck tumor
2012	[58]	Gurley et al.	Human skeletal muscle	BFI, hemoglobin, StO_2_ oxygen consumption rate	Handgrip exercise
2012	[11]	Shang et al.	Human skeletal muscle	BFI, hemoglobin, StO_2_ oxygen consumption rate	Fibromyalgia
2013	[59]	Buckley et al.	Human infant brain	BFI, StO_2_ oxygen consumption rate	Cardiac surgery
2013	[60]	Mesquita et al.	Human skeletal muscle	BFI, StO_2_	Peripheral artery disease
2013	[61]	Mesquita et al.	Human brain	BFI, hemoglobin oxygen consumption rate	Transcranial magnetic stimulation
2013	[62]	Li et al.	Human skeletal muscle	BFI, hemoglobin change	Cuff occlusion on upper limb
2013	[63]	Shang et al.	Human skeletal muscle	BFI, hemoglobin change	Electrical stimulation
2014	[64]	Jain et al.	Human infant brain	BFI, StO_2_ oxygen consumption rate	Congenital heart disease
2014	[65]	Cheng et al.	Human brain	BFI, hemoglobin change	Posture change
2014	[6]	Hou et al.	Human brain	BFI, hemoglobin change	Sleep apnea
2015	[66]	Henry et al.	Human skeletal muscle	BFI, hemoglobin, StO_2_ oxygen consumption rate	Plantar flexion exercise
2016	[12]	Dong et al.	Human tumor	BFI, hemoglobin change	Head/neck tumor
2017	[67]	Baker et al.	Human skeletal muscle	BFI, hemoglobin, StO_2_ oxygen consumption rate	Peripheral artery disease
2018	[68]	Ko et al.	Swine brain	BFI, hemoglobin, StO_2_ oxygen consumption rate	Deep hypothermic
